# RETURN TO SPORTS FOLLOWING KNEE OSTEOTOMY IN COMPETITIVE ATHLETES – CASE SERIES

**DOI:** 10.1590/1413-785220253301e278744

**Published:** 2025-02-03

**Authors:** Daniel Meirelles, Alexandre Carneiro Bitar, Caio D’Elia, Guilherme Garofo, Alberto Terrível, Wagner Castropil

**Affiliations:** 1 Instituto Vita, Department of Sports Medicine, São Paulo, SP, Brazil

**Keywords:** Knee, Osteotomy, Sports, Athletes, Return to Sport, Joelho, Osteotomia, Esportes, Atletas, Volta ao Esporte

## Abstract

**Objectives:**

To analyze the return to the sport and the level of sports practice in a longitudinal cohort of athletes treated with osteotomy around the knee.

**Methods:**

Active athletes who underwent osteotomy or knee surgery to treat knee osteoarthritis were included, and their data was collected retrospectively. The primary outcomes were maximum physical activity level before and after the surgery (Tegner score), time to return to maximum activities and reoperation.

**Results:**

Twenty athletes with a mean age of 33 years at the time of surgery (standard deviation 8.9 (SD)) and with a mean follow-up of 9.8 years (SD 4) were included. The mean maximum Tegner score achieved before surgery was 8.6 (SD 1.4). Nineteen patients returned to sports (95%), and 13 returned to the same prior level (65%). The median time to return to the maximum level was 13 months (mean 17.9, SD 12.4). The mean maximum postoperative Tegner score was 7.5 (SD 2.0), slightly lower than the maximum achieved before surgery (mean difference:1.1, CI:0.2-1.9, P=0.026).

**Conclusion:**

The results of this study suggest that, after osteotomies around the knee, athletes present a high rate of return to sports activities, with most returning at the same level as before the surgery. **Level of Evidence IV; Case series.**

## Resumo

**Objetivos:**

Analisar o retorno ao esporte e o nível de prática esportiva em uma coorte longitudinal de atletas tratados com osteotomia ao redor do joelho.

**Métodos:**

Foram incluídos atletas ativos submetidos à cirurgia de osteotomia ao redor do joelho para tratamento de osteoartrite do joelho e seus dados foram coletados retrospectivamente. Os desfechos primários foram nível máximo de atividade física antes e depois da cirurgia (escore de Tegner), tempo para retorno às atividades máximas e reoperação.

**Resultados:**

Foram incluídos 20 atletas com idade média de 33 anos no momento da cirurgia (desvio padrão 8,9 (DP)) e com seguimento médio de 9,8 anos (DP 4). A média do escore máximo de Tegner alcançado antes da cirurgia foi de 8,6 (DP 1,4). Dezenove pacientes retornaram à prática esportiva (95%) e 13 retornaram ao mesmo nível anterior (65%). O tempo mediano para retornar ao nível máximo foi de 13 meses (média 17,9, DP 12,4). A média do escore máximo de Tegner pós-operatório foi de 7,5 (DP 2,0), ligeiramente inferior ao máximo alcançado antes da cirurgia (diferença média: 1,1, IC: 0,2-1,9, P = 0,026).

**Conclusão:**

Os resultados deste estudo sugerem que, após osteotomias ao redor do joelho, os atletas apresentam alto índice de retorno às atividades esportivas, sendo que a maioria retorna no mesmo nível de antes da cirurgia. **Nível de Evidência IV; Serie de Casos.**

## INTRODUCTION

Osteoarthritis (OA) of the knee is often observed in high-demand athletes. OA patients often suffer from pain, limitations on activities, and worsening quality of life.^1^ Initially, the treatment is conservative, through behavioral measures, such as weight loss, and pharmacological ones aimed at reducing disease progression and improving limb function.^2^

While total arthroplasty of the knee is reserved for elderly and less active patients with OA, osteotomies around the knee are indicated for young, active individuals, who still fully exercise their physical capacity.^3^ Treatment in these patients, in addition to aiming to relieve pain, is focused on returning to sports activities and slowing down the progression of the disease. According to the literature, the candidates for osteotomy around the knee are patients under 60 years of age, with unicompartmental OA, without ligament instability, and with a good range of knee motion.^4^,^5^ Other procedures associated with osteotomy can be performed, such as meniscal transplant, cartilage repair procedures, or even ligament reconstruction.^1^,^6^

In recent years, the improvement in surgical techniques, the fixation materials available, and the increase in evidence in the literature have led to better postoperative results in terms of function and pain, and to the longevity of the procedure. Survival rates for high tibial osteotomy at five and ten years are 87-99% and 66-84%, respectively,^7^,^8^,^9^ and for distal femoral osteotomy are 75-90% at five years and 64-82% at ten years.^10^,^11^,^12^,^13^

Bonnin et al. concluded that only 56% of the patients submitted to osteotomy around the knee were able to return to the preoperative sports level and that 62% had limited activity due to the operated knee.^14^ Although the literature about the results from osteotomies around the knee to treat OA is well established, few studies have considered a population of athletes who play highly demanding sports.^15^,^16^

The objective of the study was to analyze the time to return to the sport and the sports practice level of a longitudinal cohort of athletes who underwent osteotomy around the knee at a single center.

## MATERIALS AND METHODS

### Study design

This was a retrospective case series study, conducted at a single center, which included patients who underwent surgery between January 2000 and December 2014. This study was evaluated and approved by the Research Ethics Committee under the number 4.160.318. The authors declare that there is no conflict of interest that interferes with this study.

### Study population

Inclusion criteria: to have undergone osteotomy around the knee surgery for the treatment of osteoarthritis of the knee associated or not with ligament reconstructions (the other reconstruction procedures were not evaluated in this study and were not exclusion criteria) , to play sports at a competitive level and be in an active career stage. Exclusion criteria: incomplete medical record data or inability to contact the participant for data collection. All the surgeries were performed by the senior surgeons at our institution. Cases treated with corrective osteotomies of femur and tibia (axial osteotomies) or Fulkerson osteotomy with or without ligament reconstruction, cartilage repair and/or meniscal procedures were included.

### Data collection and analysis

The following data were collected: sex, age at the time of surgery, maximum level of physical activity prior to surgery as measured by the Tegner score, type of surgery performed, return to sports, need to change sport after surgery, time to return to athletic activity (at least four times a week), time to return to activity at the maximum level achieved, maximum physical activity level (Tegner) after surgery, current physical activity level (Tegner) (maximum postoperative follow-up), reoperations, and an evaluation of expectations.^17^ We also analyzed the results according to type of osteotomy.

An evaluator who did not participate in the surgeries collected and analyzed the data. Initially, a thorough search of the medical records was conducted. Missing data were completed directly by the participants using a digital questionnaire. Analysis was conducted with qualitative and quantitative descriptions of the data. The Wilcoxon signed rank test was used to compare the pre- and postoperative maximum activity levels. An analysis by subgroup was performed for the different types of osteotomy: axial osteotomies (tibial and femoral) and Fulkerson osteotomies. The level of significance adopted was 95% and the tests were performed using SPSS software.

## RESULTS

Of the 26 initially eligible individuals found in the search of the medical records of our institution, we were able to include 20 athletes in this study (77% retention) with an average of ten years of postoperative follow-up. The baseline data are shown in Table 1.

**Table 1:** Demographic data.

Patients	20
Females / Males	6 (30%)/ 14 (70%)
Age (years)	33 ± 9 (20-57)
Follow-up after osteotomy (years)	10 ± 4 (5-20)
Maximum Tegner score preoperatively	8.6 ± 1.4 (6-10)

Data are shown as mean ± standard deviation (minimum - maximum) or as observed absolute values (percentage among total cases)

Among the surgical procedures of the 20 included patients, 14 were axial osteotomies (ten opening wedge high tibial valgus, one femoral extension, and three opening wedge distal femoral varus) and six were Fulkerson anterior tibial tubercle osteotomies.

After surgery, 19 patients returned to sports (95% of cases), 13 of whom returned to the same previous level (65%). Four individuals changed sports after surgery (20%). The median postoperative time for return to athletic activities (at least four times a week) was nine months (mean 11.7, SD 9.0) and the median postoperative time for return to the maximum level was 13 months (mean 7.9, SD 12.4).

The mean maximum postoperative Tegner score was 7.5 (SD 2.0) and was slightly lower than the mean of the maximum scores that had been achieved during the sports career prior to surgery (mean of differences: 1.1, CI 0.2-1.9, *P*=0.026), Table 2.

**Table 2:** Clinical outcomes.

	Total (n=20)	Axial (n=14)	Fulkerson (n=6)	P value (Axial vs Fulkerson)
Tegner score preoperatively	8.6 ± 1.4	8.6 ± 1,3	8.5 ± 1.6	0.891
Tegner score postoperatively	7.5 ± 2.0	7.4 ± 2.2	7.8 ± 1.6	0.854
P value (pre vs postop)	0.026*	0.042*	0.317	-
Return to the preop level	65%	64%	83%	0.612
Reoperation	15%	14%	33%	0.342

Data are shown as mean ± standard deviation (minimum - maximum) or as observed absolute values (percentage among total cases). Tegner scores shown represent the maximum level reached both pre and postoperatively. *Statistically significant (*P*<0.05)

The patients were able to maintain this maximum level following surgery for an average survival time of 5.1 years (SD 3.7). At the final clinical follow-up of ten years, the individuals reported a mean Tegner score of 6.2 (SD 1.8) (Table 1). Three patients (15%) underwent reoperations for removal of synthesis material related to the osteotomies performed (after two, 18 and 24 months).

Even though the patient sample was small, we observed a better return to maximum level in the patients submitted to Fulkerson osteotomies than in the patients who underwent axial osteotomies, but with greater need for removal of the synthesis implant, but given the sample (n = 6) of Fulkerson osteotomy, we cannot consider this as a tendency, but only a random finding that must be proven with a greater subject number.

## DISCUSSION

Studies in the population that practiced recreational activities and sports have shown that young, active patients submitted to knee osteotomy were able to return to sports activities in a similar level as before surgery.^18^,^19^,^20^ Bonnin et al. concluded that young, motivated patients are able to return to high demand sports activities, which corroborates our results, since we demonstrated a high rate of return to sports: 95% of the high demand athletes returned to sports, with 65% achieving their preoperative sports level.^14^

We also chose to compare the maximum preoperative and postoperative performance levels of the athletes. The mean physical activity level achieved after surgery was very close to that achieved by the athletes before the need for treatment. De Carvalho et al. identified a mean Tegner score of 3.0 (range 1-7) both before and after the surgical procedure.^21^ Hoorntje et al. evaluated the Tegner score the same way that we approached it in the present study and arrived at a mean result of four, prior to symptoms, and of three, postoperatively.^22^ We can see that, because the cohort analyzed in our study was composed of competitive-level athletes, we reached mean maximum Tegner score values of 7.5 following surgery, slightly lower than the maximum Tegner score that the athletes had achieved in their career at any time prior to surgery (mean of 8.6). Another important fact was that our sample was composed of high demand athletes, almost all of them at a competitive level, including Olympic athletes. We observed very high return to sports rates as compared to those reported to date in the literature. Hoorntje et al. obtained a postoperative rate of return to sports of 82%, but in a cohort without competitive-level athletes.^23^ Kanto et al. studied 77 patients with Tegner scores ≥ 5 points before surgery and a mean age of 56.1 ± 11.6 years (range 26–79) and confirmed a 75.3% return rate to the same level in a mean time to return of 8.7 ± 2.7 months (range 6–14).^24^

Over the last decade, several studies have focused on demonstrating the rate of return to sports in patients submitted to knee osteotomy. Older studies reported a rate of return to sports following knee osteotomy below 50%.^25^ With the improvement in surgical techniques for fixation in osteotomies, such as fixed angle plates, surgical outcomes have undergone an important evolution with a significant increase in patients who returned to sports after undergoing osteotomies around the knee.^20^,^25^ The percentage of patients (95%) who returned to sports following the surgical procedure in our study, was higher than that found in two recent systematic literature reviews.^22^,^26^ If we analyze studies in the literature that only considered return to high-impact activities, we find rates from 35 to 70%.^20^,^26^,^27^

Regarding the time to return to sports activities following the surgical procedure, Hoorntje et al. concluded that 75% of the patients who returned to sports did so after less than six months.^23^ In another study, the same author reported that 71% of the patients returned to playing sports in less than six months, with 50% returning less than 15 weeks after distal femoral osteotomy.^28^ Jacquet et al. reached a similar outcome in which the patients who underwent high tibial osteotomy returned to sports practice in an average of 4.9 months.^29^ In our study, we had a longer time to return to sports, at nine months on average. Our hypothesis for this finding is that many of the patients did not undergo only osteotomy around the knee. Most of them had associated ligament or cartilage repair procedures, which increased the time to return. Studies of osteotomies around the knee associated with meniscal transplants had mean return to sports times of 16.9 months^30^ and 9.7 months.^31^

If we consider only the patients who were submitted to anteromedialization osteotomy of the anterior tibial tubercle (ATT), 86% of this group returned to the same athletic level as before the onset of symptoms. This finding is in line with that published by Liu et al., who evaluated the return to sports after ATT osteotomy in a group of 48 patients, 83.3% of whom returned to playing sports.^30^ However, the group was not composed of high-demand athletes. Finally, we also decided to conduct an analysis grouping the tibial and femoral osteotomies (axial osteotomies) and comparing their results with the Fulkerson osteotomy results. Even though the patient sample was small, we observed a better return to maximum level in the patients submitted to Fulkerson osteotomies than in the patients who underwent axial osteotomies, but with a greater need for removal of synthesis material. Neither showed a statistical difference. Because of the subjective limitation, it is possible that it could not be a tendency, but only a coincidence, which is necessary to be clarified in future studies with a greater number of subjects. Tjoumakaris et al. performed Fulkerson osteotomy and lateral retinacular release in athletes due to patellofemoral instability.^32^ All 34 patients returned to sports practice and 17 had to have the osteotomy fixation screws removed after eight months.

It is well established in literature, the use of osteotomy for the axis correction in patients not only with osteoarthritis but also chondral lesions, in association with other procedures for chondral repair. In this study, we have chosen only patients with established osteoarthritis, given the difference of both diseases.^33^,^34^

The current study has some important limitations. First, the study is retrospective and subject to the inherent limitations of this design, with patients being asked questions about events that occurred, in some cases, many years before. In addition, the sample size is relatively small and thus, subgroup analyses, such as the comparison between the results of patients submitted to axial osteotomy and of patients submitted to ATT osteotomy, or between prognostic factors, end up having little statistical power. Another limitation of this study is that different osteotomy around the knee techniques were used by different surgeons, and we did not analyze the degrees of correction of the osteotomies, directly related to their success. Also, we did not analyze the use of other procedures combined to the osteotomies such as meniscus sutures, ligament reconstructions, which could have influenced the patient’s final results.

## CONCLUSION

The study suggests that osteotomies around the knee may be valid treatments for athletes of competitive age who want to return to sports activities. These results also show that in some cases it is possible to return to practically the same sports level that was achieved by the athlete at their peak, prior to surgery. It should be emphasized that a thorough analysis of each case and the use of a pertinent surgical technique are essential for safe treatment and for greater chances of reaching the individual goal. Multicenter studies that cover a greater number of athletes may be able to identify the best prognostic factors in these clinical situations.

## References

[1] Brouwer RW, Huizinga MR, Duivenvoorden T, van Raaij TM, Verhagen AP, Bierma-Zeinstra SM (2014). Osteotomy for treating knee osteoarthritis. Cochrane Database Syst Rev.

[2] Fibel KH, Hillstrom HJ, Halpern BC (2015). State-of-the-Art management of knee osteoarthritis. World J Clin Cases.

[3] Kohn L, Sauerschnig M, Iskansar S, Lorenz S, Meidinger G, Imhoff AB (2013). Age does not influence the clinical outcome after high tibial osteotomy. Knee Surg Sports Traumatol Arthrosc.

[4] Prodromos CC, Amendola A, Jakob RP (2015). High tibial osteotomy: indications, techniques, and postoperative management. Instr Course Lect.

[5] Khan M, Evaniew N, Bedi A, Ayeni OR, Bhandari M (2014). Arthroscopic surgery for degenerative tears of the meniscus: A systematic review and meta-analysis. CMAJ.

[6] Savarese E, Bisicchia S, Romeo R, Amendola A (2011). Role of high tibial osteotomy in chronic injuries of posterior cruciate ligament and posterolateral corner. J Orthop Traumatol.

[7] Niinimäki TT, Eskelinen A, Mann BS, Junnila M, Ohtonen P, Leppilahti J (2012). Survivorship of high tibial osteotomy in the treatment of osteoarthritis of the knee: Finnish registry-based study of 3195 knees. J Bone Joint Surg Br.

[8] Bode G, von Heyden J, Pestka J, Schmal H, Salzmann G, Südkamp N (2015). Prospective 5-year survival rate data following open-wedge valgus high tibial osteotomy. Knee Surg Sports Traumatol Arthrosc.

[9] Efe T, Ahmed G, Heyse TJ, Boudriot U, Timmesfeld N, Fuchs-Winkelmann S (2011). Closing-wedge high tibial osteotomy: Survival and risk factor analysis at long-term follow up. BMC Musculoskelet Disord.

[10] Saithna A, Kundra R, Getgood A, Spalding T (2014). Opening wedge distal femoral varus osteotomy for lateral compartment osteoarthritis in the valgus knee. Knee.

[11] Cameron JI, McCauley JC, Kermanshahi AY, Bugbee WD (2015). Lateral Opening-wedge Distal Femoral Osteotomy: Pain Relief, Functional Improvement, and Survivorship at 5 Years. Clin Orthop Relat Res.

[12] Backstein D, Morag G, Hanna S, Safir O, Gross A (2007). Long-Term Follow-Up of Distal Femoral Varus Osteotomy of the Knee. J Arthroplasty.

[13] Sternheim A, Garbedian S, Backstein D (2011). Distal femoral varus osteotomy: Unloading the lateral compartment: Long-term follow-up of 45 medial closing wedge osteotomies. Orthopedics.

[14] Bonnin MP, Laurent JR, Zadegan F, Badet R, Pooler Archbold HA, Servien E (2013). Can patients really participate in sport after high tibial osteotomy?. Knee Surg Sports Traumatol Arthrosc.

[15] Uquillas C, Rossy W, Nathasingh CK, Strauss E, Jazrawi L, Gonzalez-Lomas G (2014). Osteotomies about the knee: AAOS exhibit selection. J Bone Joint Surg Am.

[16] Gao L, Madry H, Chugaev DV, Denti M, Frolov A, Burtsev M (2019). Advances in modern osteotomies around the knee: Report on the Association of Sports Traumatology, Arthroscopy, Orthopaedic surgery, Rehabilitation (ASTAOR) Moscow International Osteotomy Congress 2017. J Exp Orthop.

[17] Tegner Y, Lysholm J (1985). Rating systems in the evaluation of knee ligament injuries. Clin Orthop Relat Res.

[18] Holden D, James S, Larson R, Slocum D (1988). Proximal Tibial Osteotomy in Patients Who Are Fifty Years Old or Less. A Long-term Follow-Up Study. J Bone Joint Surg Am.

[19] Korovessis P, Katsoudas G, Salonikides P, Stamatakis M, Baikousis A (1999). Medium- and long-term results of high tibial osteotomy for varus gonarthrosis in an agricultural population. Orthopedics.

[20] Salzmann GM, Ahrens P, Naal FD, El-Azab H, Spang JT, Imhoff AB (2009). Sporting activity after high tibial osteotomy for the treatment of medial compartment knee osteoarthritis. Am J Sports Med.

[21] de Carvalho LH, Temponi EF, Soares LFM, Gonçalves MBJ, Costa LP (2014). Physical activity after distal femur osteotomy for the treatment of lateral compartment knee osteoarthritis. Knee Surg Sports Traumatol Arthrosc.

[22] Hoorntje A, Witjes S, Kuijer PPFM, Koenraadt KLM, van Geenen RCI, Daams JG (2017). High Rates of Return to Sports Activities and Work After Osteotomies Around the Knee: A Systematic Review and Meta-Analysis. Sports Med.

[23] Hoorntje A, Kuijer PPFM, van Ginneken BT, Koenraadt KLM, van Geenen RCI, Kerkhoffs GMMJ (2019). Prognostic Factors for Return to Sport After High Tibial Osteotomy: A Directed Acyclic Graph Approach. Am J Sports Med.

[24] Kanto R, Nakayama H, Iseki T, Onishi S, Ukon R, Kanto M (2021). Return to sports rate after opening wedge high tibial osteotomy in athletes. Knee Surg Sports Traumatol Arthrosc.

[25] Lerat JL, Moyen B, Garin C, Mandrino A, Besse JL, Brunet-Guedj E (1993). Anterior laxity and internal arthritis of the knee. Results of the reconstruction of the anterior cruciate ligament associated with tibial osteotomy. Rev Chir Orthop Reparatrice Appar Mot.

[26] Ekhtiari S, Haldane CE, De Sa D, Simunovic N, Musahl V, Ayeni OR (2016). Return to work and sport following high tibial osteotomy: A systematic review. J Bone Joint Surg Am.

[27] Johnstone SF, Tranovich MJ, Vyas D, Wright VJ (2013). Unicompartmental arthritis in the aging athlete: Osteotomy and beyond. Curr Rev Musculoskelet Med.

[28] Hoorntje A, van Ginneken BT, Kuijer PPFM, Koenraadt KLM, van Geenen RCI, Kerkhoffs GMMJ (2019). Eight respectively nine out of ten patients return to sport and work after distal femoral osteotomy. Knee Surg Sports Traumatol Arthrosc.

[29] Jacquet C, Gulagaci F, Schmidt A, Pendse A, Parratte S, Argenson JN (2020). Opening wedge high tibial osteotomy allows better outcomes than unicompartmental knee arthroplasty in patients expecting to return to impact sports. Knee Surg Sports Traumatol Arthrosc.

[30] Liu JN, Wu HH, Garcia GH, Kalbian IL, Strickland SM, Shubin Stein BE (2018). Return to Sports After Tibial Tubercle Osteotomy for Patellofemoral Pain and Osteoarthritis. Arthrosc.

[31] Liu JN, Agarwalla A, Garcia GH, Christian DR, Gowd AK, Yanke AB (2019). Return to Sport and Work After High Tibial Osteotomy With Concomitant Medial Meniscal Allograft Transplant. Arthrosc.

[32] Tjoumakaris FP, Forsythe B, Bradley JP (2010). Patellofemoral instability in athletes: Treatment via modified fulkerson osteotomy and lateral release. Am J Sports Med.

[33] Dhillon J, Kraeutler MJ, Fasulo SM, Belk JW, Scillia AJ, McCulloch PC (2023). Isolated Osteotomy Versus Combined Osteotomy and Cartilage Repair for Osteoarthritis or Focal Chondral Defects of the Medial Compartment of the Knee Joint: A Systematic Review. Orthop J Sports Med.

[34] Ghasemi SA, Kolesnick E, Murray BC, Leiby BE, Bartolozzi AR, Zaslav KR (2024). High tibial osteotomy combined with cartilage restoration: A systematic review of clinical outcomes and prognostic factors. J Clin Orthop Trauma.

